# Impact of in-hospital body mass index variation on 28-day mortality in critically ill surgical patients: a multi-center retrospective analysis

**DOI:** 10.3389/fmed.2025.1738654

**Published:** 2026-01-27

**Authors:** Bocheng Yang, Gang Xu, Jiagui Zhao, Xiaoan Yang, Qinghe Huang

**Affiliations:** 1 Division of Plastic Surgery, Zhongshan Hospital Xiamen University, School of Medicine, Xiamen University, Xiamen, China; 2 Medical Emergency Center of Xiamen, Xiamen, China; 3 Department of Critical Care Medicine, Zhongda Hospital, School of Medicine, Southeast University, Nanjing, Jiangsu, China; 4 Department of Infectious Diseases, The Third Affiliated Hospital, Sun Yat-sen University, Guangzhou, China; 5 Department of ICU, Zhongshan Hospital of Xiamen University, School of Medicine, Xiamen University, Xiamen, China

**Keywords:** 28-day mortality, BMI change, emergency department admission, surgical ICU, U-shaped relationship

## Abstract

**Background:**

The prognostic value of dynamic body mass index (BMI) changes during hospitalization in surgical intensive care unit (ICU) patients admitted emergently remains unclear. This study aimed to investigate the association between in-hospital BMI change and 28-day mortality in this high-risk population.

**Methods:**

This retrospective cohort study utilized data from the eICU Collaborative Research Database (2014–2015). A total of 20,543 adult surgical ICU patients admitted via the emergency department (ED) were included. BMI change was calculated as discharge BMI minus admission BMI. Multivariable Cox regression, restricted cubic splines, and subgroup analyses were employed to evaluate the association between BMI change and mortality.

**Results:**

The 28-day ICU mortality was 4.70%. BMI change exhibited a U-shaped, non-linear association with death: risk declined modestly as BMI rose toward the nadir of −1.75 kg/m^2^, then increased sharply thereafter. Each additional kg/m^2^ above this threshold raised mortality by 9% (HR 1.09, 95% CI 1.05–1.12, *p* < 0.0001). Patients in the highest BMI-gain quartile faced a 52% higher risk than those in the lowest quartile (HR 1.52, 95% CI 1.27–1.82, p < 0.0001). Dynamic BMI change outperformed static BMI or weight measures (AUC 57.9).

**Conclusion:**

In-hospital BMI change is a significant predictor of 28-day mortality in surgical ICU patients admitted via the ED. A moderate reduction in BMI (−1.75 kg/m^2^) was associated with the lowest mortality risk. Dynamic BMI monitoring may enhance risk stratification and guide personalized fluid management in this population.

## 1 Introduction

Critically ill patients admitted to the intensive care unit (ICU) from the emergency department (ED) represent a population at high risk of adverse outcomes, with mortality rates significantly influenced by the timeliness and appropriateness of initial management (1, 2). Among these, patients requiring surgical ICU admission face unique challenges due to the interplay of acute surgical stress, underlying comorbidities, and the metabolic consequences of critical illness (3, 4). The ED is a major source of ICU admissions, with surgical ICU mortality rates ranging from 1.3 to 7.56% (5–7). Traditional prognostic tools, such as the Acute Physiology and Chronic Health Evaluation (APACHE) IV and the Sequential Organ Failure Assessment (SOFA) score (8, 9), though valuable, often incorporate parameters that are not readily available during the initial ED assessment, limiting their utility for refining prognosis and guiding subsequent fluid-nutrition management.

Body mass index (BMI), the cornerstone of current obesity classification systems, is widely used to reflect nutritional and metabolic status because of its simplicity and broad availability (10). The relationship between BMI and critical-care outcomes has been extensively studied, often manifesting as an “obesity paradox,” in which overweight or mildly obese patients experience lower mortality than underweight or normal-weight individuals (11–13). However, the vast majority of this evidence is based on a single, static BMI measurement obtained at hospital admission. This static snapshot fails to capture the dynamic physiological changes that occur during hospitalization, such as fluid shifts, catabolic states, and the effects of nutritional support, all of which can significantly alter body composition and potentially influence prognosis (14).

This is particularly relevant in surgical ICU populations. The metabolic response to surgical trauma and critical illness can lead to rapid changes in weight, reflecting either fluid retention or lean body mass catabolism (15, 16). In the acute phase of critical illness, particularly in surgical patients, changes in body weight and BMI are predominantly influenced by fluid shifts and balance, rather than changes in fat or lean mass. Thus, to some extent, an increase in BMI can be regarded as a surrogate for fluid overload—a well-established risk factor for organ dysfunction and increased mortality in the ICU (17). Conversely, a decrease in BMI might reflect inadequate caloric intake or severe catabolism, also potentially leading to poorer outcomes (18). Despite the biological plausibility of these mechanisms, the prognostic value of dynamic, in-hospital BMI change has not been thoroughly investigated in surgical patients admitted to the ICU from the emergency setting.

To address this significant knowledge gap, we conducted a large, retrospective cohort study utilizing the eICU Collaborative Research Database (19). Our primary objective was to investigate the association between the change in BMI during hospitalization and 28-day all-cause mortality in a cohort of surgical ICU patients admitted from the ED. We further aimed to explore the nature of this association (linear vs. non-linear) and to evaluate whether dynamic BMI change offers superior prognostic value compared to static admission or discharge BMI measurements.

## 2 Methods

### 2.1 Study design and data source

This retrospective multicenter cohort study utilized data from the eICU Collaborative Research Database (eICU-CRD), a multi-center ICU database comprising de-identified clinical data from 208 hospitals across the United States between 2014 and 2015 (19). The database includes high-granularity clinical variables collected from electronic health records, including vital signs, laboratory results, medication records, and outcomes, making it suitable for investigating dynamic clinical changes such as BMI variation during hospitalization.

### 2.2 Ethical statement

The study was conducted in accordance with the ethical principles of the Declaration of Helsinki. The use of the eICU-CRD was approved by the Institutional Review Board (IRB) of the Massachusetts Institute of Technology (MIT), which waived the requirement for informed consent due to the retrospective and de-identified nature of the data (Record ID: 66836126).

### 2.3 Study population

**Figure 1:**
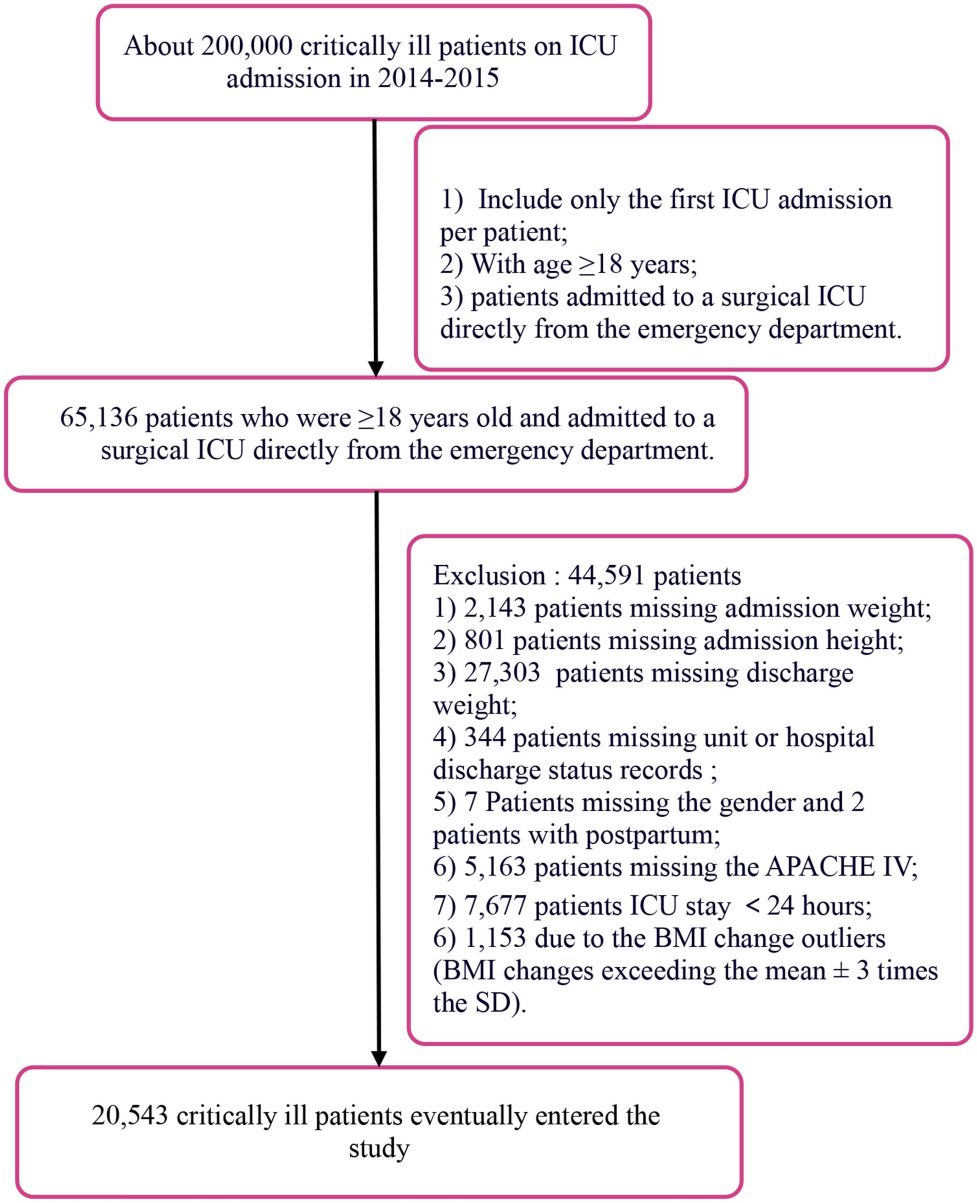
Flow chart of study population.

We conducted a retrospective, multicenter cohort study using the eICU Collaborative Research Database (2014–2015). The patient selection process is detailed in Figure 1. From an initial pool of approximately 200,000 critically ill adults, we identified 65,136 patients aged ≥18 years who were admitted to a surgical ICU directly from the ED. After applying the inclusion and exclusion criteria, 44,591 patients were excluded for the following reasons: missing admission weight (*n* = 2,143), missing admission height (*n* = 801), missing discharge weight (*n* = 27,303), missing unit or hospital discharge status records (*n* = 344), missing gender information or postpartum status (*n* = 9), missing APACHE IV score (*n* = 5,163), ICU length of stay <24 h (*n* = 7,677), and BMI change outliers (exceeding mean ± 3 SD, *n* = 1,153) (20). After these exclusions, a total of 20,543 critically ill patients were included in the final analysis.

### 2.4 Exposure variables and outcome

Exposure variable: The primary exposure was the change in BMI during hospitalization, calculated as: BMI change = BMI at discharge – BMI in admission. Both admission BMI were calculated using weight and height recorded within the first 24 h of hospital stay. Discharge weight was defined as the last available weight measurement documented in the electronic health record prior to ICU discharge or death. The exact timing and frequency of routine weight measurements were not standardized across participating ICUs and are not captured in the eICU-CRD. Consequently, the interval between the last weight measurement and the endpoint event (discharge or death) varies among patients.

### 2.5 Outcome

The primary outcome of this study was 28-day ICU mortality, defined as death from any cause occurring within 28 days of ICU admission.

### 2.6 Covariates

We collected the following covariates based on clinical relevance and previous literature: ① Demographics: age, gender, ethnicity; ② Clinical scores: APACHE IV, SOFA, GCS score; ③ Laboratory parameters: BUN, serum creatinine (Scr), aspartate aminotransferase (AST); alanine aminotransferase (ALT), platelets (PLT), white blood cell count (WBC), red cell distribution width (RDW); ④ Comorbidities: diabetes mellitus (DM), chronic obstructive pulmonary disease (COPD), congestive heart failure (CHF), acute myocardial infarction (AMI); ⑤ ICU length of stay (LOS).

### 2.7 Statistical analysis

Continuous variables were expressed as mean ± standard deviation (SD) or median with interquartile range (IQR), as appropriate; categorical variables were presented as frequencies and percentages. Differences across BMI-change quartiles were compared using one-way ANOVA or the Kruskal–Wallis test for continuous variables, and the χ^2^ test for categorical variables.

The association between BMI change and 28-day ICU mortality was assessed using multivariable Cox proportional hazards regression. To avoid collinearity and over-adjustment, we first calculated variance inflation factors (VIF) and excluded variables with VIF > 5. The remaining candidates were subjected to LASSO regression (10-fold cross-validation, λ1SE criterion), yielding a parsimonious covariate set: Age, ethnicity, gender, ICU length of stay, vent, SOFA and APACHE IV. These seven variables constituted the final Adjust III model. Three sequential models were constructed: Model I—unadjusted; Model II—adjusted for demographics (age, gender, ethnicity); Model III—fully adjusted for demographics, clinical scores (Age, ethnicity, gender, ICU length of stay, vent, SOFA and APACHE IV.

To explore potential non-linear relationships, we performed restricted cubic spline (RCS) analyses with four knots located at the 5th, 35th, 65th and 95th percentiles of BMI change; a likelihood-ratio test compared the linear and non-linear models (21). Kaplan–Meier curves and log-rank tests were used to illustrate 28-day survival across BMI-change quartiles.

Subgroup analyses were conducted according to age (<65 vs. ≥ 65 years), gender, and presence of comorbidities, with interaction terms tested to evaluate effect modification. Missing data is inevitable in observational studies. In this research, missing data percentages were as follows: age at 0.00% (0 case), BMI at 0.00% (0 case), gender at 0.00% (0 case), GCS score at 0.56% (115 case), BNU at 9.56% (1964 case), serum calcium at 13.7% (2,814 case), GLU at 9.51% (1953 case), Scr at baseline at 9.38% (1927 case), serum K at 8.95 (1838 case), PLT at 14.32% (2,942 case), RBC at 14.50% (2,979 case), HBG at 13.23% (2,717 case), RDW at 18.35% (3,770 case), and WBC at 14.10% (2,897 case). To address this issue, we utilized multiple imputation. This process assumed that the missing data were Missing at Random (MAR) (22). No significant differences in baseline characteristics after imputation (Supplementary Table S1). Receiver operating characteristic (ROC) curve analysis was conducted to calculate the area under the curve (AUC) and optimal thresholds to demonstrate the predictive capability of the BMI change. A two-tailed alpha level was set at 0.05. All statistical analyses were conducted using EmpowerStats^1^ (X&Y Solutions, Inc., Boston, MA) and R software version 3.6.1^2^.

## 3 Results

### 3.1 Characteristics of participants

As showed in Tables 1, a total of 20,543 participants were included in this study, with 10,869 (52.91%) males and 9,674 (47.09%) females. The mean age was 62.51 years. The 28-day mortality rate in the surgical ICU is 4.7%; however, we found 708 patients died after being transferred out of the ICU. The BMI change exhibited an approximately normal distribution, with values ranging from −7.54 kg/m^2^ to 7.87 kg/m^2^. The mean change in BMI was 0.218 kg/m^2^ (Supplementary Figure S1).

**Table 1:** The baseline characteristics of participants.

Characteristics	Q1(−7.54−0.58)	Q2((−0.57−0.01)	Q3(−0.01–1.05)	Q4(1.06–7.87)	*p*-value
*N*	5,126	2,553	7,706	5,158	
Age(years)	62.90 ± 17.30	63.02 ± 17.40	62.05 ± 17.68	62.52 ± 17.28	0.018
Admission BMI (kg/m^2^)	30.25 ± 8.80	28.55 ± 8.02	28.49 ± 7.90	27.98 ± 8.49	<0.001
Discharge BMI (kg/m^2^)	28.17 ± 8.61	28.30 ± 8.02	28.78 ± 7.89	30.62 ± 8.68	<0.001
Gender					<0.001
Female, *n* (%)	2,285 (44.58%)	1,072 (41.99%)	3,609 (46.83%)	2,708 (52.50%)	
Male, *n* (%)	2,841 (55.42%)	1,481 (58.01%)	4,097 (53.17%)	2,450 (47.50%)	
Ethnicity					<0.001
Caucasian, *n* (%)	3,961 (77.27%)	2078 (81.39%)	6,111 (79.30%)	4,086 (79.22%)	
African-American, *n* (%)	616 (12.02%)	267 (10.46%)	872 (11.32%)	570 (11.05%)	
Hispanic, *n* (%)	226 (4.41%)	77 (3.02%)	287 (3.72%)	199 (3.86%)	
Asian, *n* (%)	132 (2.58%)	43 (1.68%)	102 (1.32%)	113 (2.19%)	
Native American, *n* (%)	60 (1.17%)	24 (0.94%)	93 (1.21%)	43 (0.83%)	
Unknown, *n* (%)	131 (2.56%)	64 (2.51%)	241 (3.13%)	147 (2.85%)	
GCS score	12.14 ± 3.84	12.94 ± 3.39	12.89 ± 3.43	12.28 ± 3.84	<0.001
APACHE IV score	58.63 ± 25.11	54.32 ± 24.08	54.07 ± 24.23	63.27 ± 27.51	<0.001
SOFA score	3.19 ± 2.70	2.71 ± 2.51	2.78 ± 2.53	3.75 ± 2.99	<0.001
Vent, *n* (%)	1,678 (32.74%)	592 (23.19%)	1,612 (20.92%)	1,418 (27.49%)	<0.001
Intubated, *n* (%)	965 (18.83%)	298 (11.67%)	882 (11.45%)	913 (17.70%)	<0.001
Dialysis, *n* (%)	230 (4.49%)	87 (3.41%)	198 (2.57%)	155 (3.01%)	<0.001
Glucocorticoid use	8 (0.16%)	4 (0.16%)	56 (0.73%)	14 (0.27%)	<0.001
Laboratory tests					
BUN, median (IQR), (mmol/L)	20.00 (13.00–33.00)	18.00 (12.00–30.00)	18.00 (12.00–30.00)	22.00 (14.00–37.00)	<0.001
Scr (mg/dL)	1.00 (0.74–1.60)	0.95 (0.72–1.40)	0.95 (0.72–1.40)	1.07 (0.77–1.78)	<0.001
Serum calcium, (mmol/L)	8.39 ± 0.77	8.46 ± 0.76	8.40 ± 0.77	8.16 ± 0.84	<0.001
Serum K, (mmol/L)	4.08 ± 0.72	4.06 ± 0.68	4.07 ± 0.68	4.10 ± 0.76	0.032
GLU, median (IQR), (mg/dl)	150.52 ± 90.63	148.33 ± 85.52	148.89 ± 92.85	157.42 ± 104.17	<0.001
AST, (U/L)	29.00 (18.00–61.00)	26.00 (17.00–52.00)	26.00 (17.00–52.00)	30.00 (18.00–71.00)	0.002
ALT, (U/L)	26.00 (16.00–46.00)	25.00 (16.00–43.00)	25.00 (16.00–42.00)	27.00 (16.00–51.00)	<0.001
PLT, (×10^9^/L)	210.14 ± 94.50	210.98 ± 94.45	208.81 ± 91.58	203.85 ± 99.55	0.001
RBC, (×10^12^/L)	3.82 ± 0.79	3.87 ± 0.77	3.83 ± 0.77	3.68 ± 0.80	<0.001
HBG, (g/L)	11.35 ± 2.40	11.53 ± 2.36	11.43 ± 2.37	10.94 ± 2.42	<0.001
RDW, (%)	15.23 ± 2.50	15.01 ± 2.34	15.03 ± 2.35	15.48 ± 2.56	<0.001
WBC, (×10^9^/L)	11.43 ± 7.02	11.62 ± 13.06	11.45 ± 10.96	12.84 ± 10.62	<0.001
Comorbidities					
COPD, *n* (%)	510 (9.95%)	261 (10.22%)	714 (9.27%)	448 (8.69%)	0.069
CHF, *n* (%)	649 (12.66%)	228 (8.93%)	561 (7.28%)	383 (7.43%)	<0.001
AMI, *n* (%)	272 (5.31%)	168 (6.58%)	433 (5.62%)	263 (5.10%)	0.05
DM, *n* (%)	1,476 (28.79%)	699 (27.38%)	1990 (25.82%)	1,442 (27.96%)	0.002
28-day ICU mortality					<0.001
No, *n* (%)	4,933 (96.23%)	2,468 (96.67%)	7,393 (95.94%)	4,783 (92.73%)	
Yes, *n* (%)	193 (3.77%)	85 (3.33%)	313 (4.06%)	375 (7.27%)	
Hospital mortality					<0.001
No, *n* (%)	4,764 (92.94%)	2,381 (93.26%)	7,181 (93.19%)	4,535 (87.92%)	
Yes, *n* (%)	362 (7.06%)	172 (6.74%)	507 525 (6.81%)	623 (12.08%)	
ICU LOS, median (IQR), d	2.53 (1.66–4.56)	2.03 (1.51–3.29)	1.97 (1.48–3.08)	2.71 (1.74–4.52)	<0.001
Hospital LOS, median (IQR), d	5.77 (3.34–9.82)	4.48 (2.82–7.16)	4.17 (2.64–6.96)	5.74 (3.54–9.41)	<0.001

Continuous variables are summarized as mean (SD) or median (trisection interval); categorical variables are presented as percentages (%). BMI, body mass index; COPD, chronic obstructive pulmonary disease; AMI, acute myocardial infarction; DM, diabetes mellitus; CHF, Congestive Heart Failure; ICU, intensive care unit; LOS, length of stay; BUN, blood urea nitrogen; RBC, red blood cell; HGB, hemoglobin; PLT, platelets; GLU, glucose; AST, aspartate aminotransferase; ALT, alanine aminotransferase; RDW, red cell distribution width; WBC, white blood cells; GCS score, Glasgow coma scale; APACHE IV, acute physiology and chronic health evaluation IV; SOFA score: sequential organ failure assessment.

After stratifying by BMI change quartiles, all baseline characteristics, except COPD and AMI, differed significantly (*p* < 0.05). Quartile 4 (Q4) had the highest discharge BMI (30.6 ± 8.7 kg/m^2^) and the highest APACHE IV and SOFA scores. RBC and Hb decreased stepwise, whereas WBC increased, consistent with a dilution–inflammation effect. Q4 also exhibited the highest 28-day ICU mortality (7.3%) and pre-discharge mortality (12.1%), along with the longest ICU stay (2.7 days), indicating that the group with the greatest BMI increase had the poorest prognosis.

### 3.2 Kaplan–Meier analysis of BMI change and 28-day mortality in ICU patients

The Log-rank test indicated a significant difference in 28-day survival among the four BMI change quartiles (*p* < 0.0001). The number at risk declined most rapidly in Q3 and Q4, suggesting that weight gain during hospitalization were strong predictors of decreased mortality (Supplementary Figure S2).

### 3.3 Factors affecting the risk of 28-day mortality were examined using univariate cox proportional hazards regression analysis

Univariate Cox regression showed that greater in-hospital BMI change was significantly associated with higher 28-day mortality (HR 1.10, 95% CI 1.08–1.13, *p* < 0.0001), independent of other significant variables (Supplementary Table S2).

### 3.4 Results from multivariate analyses using cox proportional hazards regression methods

The association between in-hospital BMI change and 28-day ICU mortality was evaluated with multivariable Cox models of increasing complexity (Table 2). Expressed continuously, each 1-unit rise in BMI was linked to higher mortality in the unadjusted model (Model I: HR 1.10, 95% CI 1.08–1.13, *p* < 0.0001). The estimate was virtually unchanged after adding demographics (Model II: HR 1.10, 95% CI 1.07–1.13, *p* < 0.0001) and attenuated only modestly after full adjustment for severity scores and laboratory variables (Model III: HR 1.06, 95% CI 1.03–1.09, p < 0.0001). When BMI change was analyzed in quartiles, a monotonic dose–response was present across all models (*P* for trend < 0.0001). In the fully adjusted model, patients in Q4 (largest BMI gain) had a 52% higher risk of death than those in Q1 (HR 1.52, 95% CI 1.27–1.82, *p* < 0.0001); Q3 also remained significant (HR 1.37, 95% CI 1.13–1.65, *p* = 0.001), whereas Q2 did not differ from Q1 (HR 1.07, 95% CI 0.82–1.39, *p* = 0.6364).

**Table 2:** Relationship between BMI change and 28-day ICU mortality in different models.

Model	Exposure	HR (95 CI) *P*-value
Model I	BMI change as continuous	1.10 (1.08, 1.13) < 0.0001
	Q1	Ref
	Q2	1.38 (1.06, 1.78) 0.0148
	Q3	1.87 (1.56, 2.24) < 0.0001
	Q4	2.07 (1.74, 2.46) < 0.0001
	P for trend	<0.0001
Model II	BMI change as continuous	1.10 (1.07, 1.13) < 0.0001
	Q1	Ref
	Q2	1.35 (1.05, 1.75) 0.0214
	Q3	1.84 (1.53, 2.20) < 0.0001
	Q4	2.04 (1.71, 2.43) < 0.0001
	P for trend	<0.0001
Model III	BMI change as continuous	1.06 (1.03, 1.09) < 0.0001
	Q1	Ref
	Q2	1.07 (0.82, 1.39) 0.6364
	Q3	1.37 (1.13, 1.65) 0.0010
	Q4	1.52 (1.27, 1.82) < 0.0001
	P for trend	<0.0001

Model I: we did not account for additional variables. Model II: we adjusted gender, age, ethnicity. Model III: we adjusted gender, age, ethnicity, SOFA score, APACHE IV score, LOS-ICU, vent. HR, Hazard Ratios; CI, confidence; Ref, reference.

Sensitivity analyses yielded consistent results across all three models: each 1 kg/m^2^ increase in BMI remained associated with a 6% higher hazard of 28-day mortality (HR 1.06; 95% CI 1.03–1.09; *p* < 0.0001). Compared with the lowest quartile, the highest quartile of BMI gain showed a 52–53% increased risk (HR ≈ 1.53; 95% CI 1.27–1.83; p < 0.0001), with a significant dose–response trend (p < 0.0001) regardless of covariate set (Table 3). Analyses repeated on the raw data set yielded materially identical findings (Supplementary Table S3), confirming robustness. These findings confirm the robustness of the primary association.

**Table 3:** Relationship between BMI change and 28-day ICU mortality in sensitivity analysis.

Model	Exposure	HR (95 CI) *P*-value
Model I	BMI change as continuous	1.06 (1.03, 1.09) < 0.0001
	Q1	1.09 (0.83, 1.41) 0.5405
	Q2	1.37 (1.13, 1.65) 0.0010
	Q3	1.53 (1.27, 1.83) < 0.0001
	Q4	
	P for trend	<0.0001
Model II	BMI change as continuous	1.06 (1.03, 1.09) < 0.0001
	Q1	Ref
	Q2	1.10 (0.84, 1.43) 0.4973
	Q3	1.38 (1.14, 1.66) 0.0008
	Q4	1.53 (1.27, 1.83) < 0.0001
	P for trend	<0.0001
Model III	BMI change as continuous	1.06 (1.03, 1.09) < 0.0001
	Q1	Ref
	Q2	1.07 (0.82, 1.39) 0.6364
	Q3	1.37 (1.13, 1.65) 0.0010
	Q4	1.52 (1.27, 1.82) < 0.0001
	P for trend	<0.0001

Model I: we adjusted gender, age, ethnicity, SOFA score, GCS score, APACHE IV score, BUN, Scr, Serum K, Serum calcium, GLU, PLT, RBC, HBG, WBC, RDW, LOS-ICU, vent, Intubated, Dialysis, Glucocorticoid use, AST and ALT. Model II: we adjusted gender, age, ethnicity, SOFA score, GCS score, APACHE IV score, BUN, Scr, Serum K, Serum calcium, GLU, PLT, RBC, HBG, WBC, RDW, LOS-ICU, vent, Intubated, Dialysis, Glucocorticoid use. Model III: we adjusted gender, age, ethnicity, SOFA score, APACHE IV score, LOS-ICU, vent.

### 3.5 Non-linear relationship between BMI change and mortality

**Figure 2:**
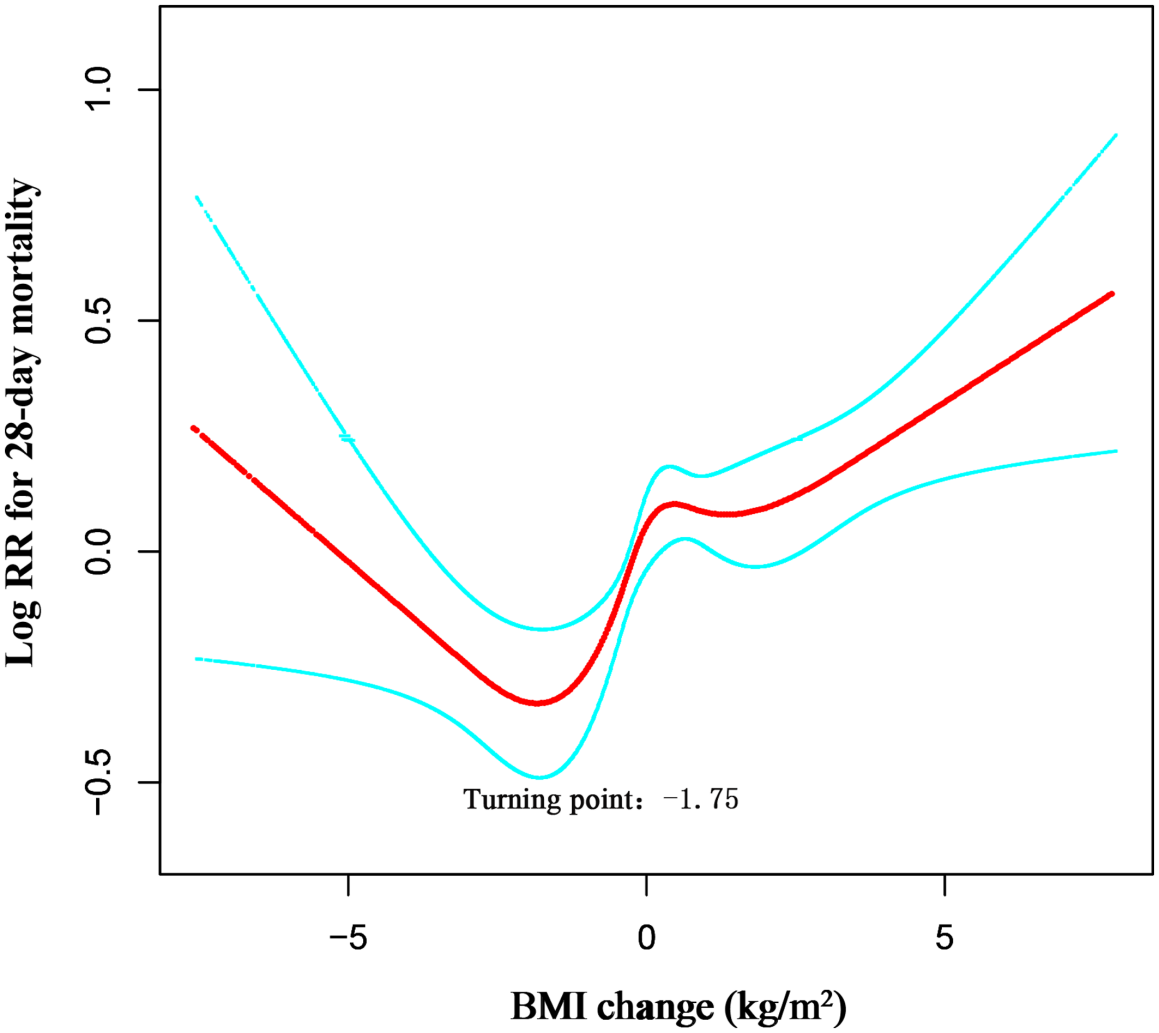
Dose–response association between absolute BMI change across adulthood and risk of all-cause mortality. Associations were examined by multivariable Cox regression models based on restricted cubic splines. Solid line represents estimates of hazard ratios and dashed line represents 95% CIs. Risk estimates were adjusted for gender, age, ethnicity, SOFA score, APACHE IV score, LOS-ICU, vent.

Restricted cubic spline analysis revealed a significant non-linear relationship between in-hospital BMI change and 28-day mortality (Overall *p* < 0.001; Non-linearity *p* < 0.001; Figure 2). Threshold analysis identified a turning point at a BMI change of −1.75 kg/m^2^ (Table 4). When BMI change was < −1.75 kg/m^2^, the association with 28-day mortality was not statistically significant (HR 0.96, 95% CI 0.86–1.04, *p* = 0.272). In contrast, for BMI change ≥ − 1.75 kg/m^2^, each 1 kg/m^2^ increase raised the risk of 28-day ICU death by 9% (HR 1.09, 95% CI 1.05–1.12, *p* < 0.0001).

**Table 4:** Threshold effect analysis of the BMI change and 28-day ICU mortality.

Models	HR (95 CI)	*p* value
One line effect	1.06 (1.03, 1.09)	<0.0001
Turning point (K)	−1.75 (kg/m^2^)	
Weight change < K	0.96 (0.86, 1.04)	0.2721
Weight change ≥ K	1.09 (1.05, 1.12)	<0.0001
*P* value for LRT test		0.023

Data were presented as HR (95 CI) *P-*value; Model I, linear analysis; Model II, non-linear analysis. Adjusted for gender, age, ethnicity, SOFA score, APACHE IV score, LOS-ICU, vent. LRT logarithm likelihood ratio test. *p* < 0.05 indicates that model II is significantly different from Model I.

The likelihood-ratio test confirmed that the non-linear model with this threshold fitted the data significantly better than a simple linear model (LRT *p* = 0.023). Thus, among surgical ICU patients admitted through the ED, the lowest 28-day mortality risk occurred at a BMI reduction of approximately 1.75 kg/m^2^; above this threshold, ICU mortality risk increased progressively with further BMI gain.

### 3.6 Predictive value of dynamic BMI change for 28-day ICU mortality in surgical ICU patients

The predictive performance of body mass metrics for 28-day mortality was evaluated using ROC curve analysis (Supplementary Figure S3). The AUC values are presented in Supplementary Table S4.

Admission weight (AUC 49.58, 95% CI 47.67–51.48%), discharge weight (AUC 51.82, 95% CI 49.92–53.72%), admission BMI (AUC 50.64, 95% CI 48.73–52.56%) and discharge BMI (AUC 52.01, 95% CI 50.11–53.90%) all exhibited poor discrimination, with areas close to 50%—equivalent to random chance.

In contrast, BMI change achieved a markedly higher AUC of 57.92% (95% CI 55.92–59.93%), indicating that the dynamic change in BMI during the ICU stay provides appreciably better, though still low-to-moderate, predictive value for 28-day mortality than any static weight or BMI measure.

### 3.7 Subgroup analyses of the association between BMI change and 28-day ICU mortality

**Figure 3:**
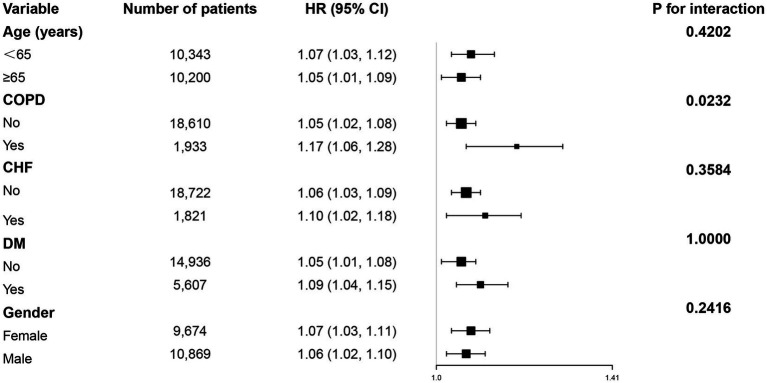
Subgroup analyses were performed to assess the association between BMI change and 28-day ICU mortality.

Subgroup analysis revealed a consistent positive association between BMI change and 28-day mortality across most predefined subgroups (Figure 3). All subgroups (including patients aged ≥65 and <65 years, with or without COPD, and of different genders) demonstrated a significant increase in mortality risk per unit increase in BMI change (all HR > 1, *p* < 0.05).

However, a significant interaction was observed only for COPD status (interaction *p* = 0.0232), indicating a potentially stronger effect of BMI change on mortality among COPD patients. No significant interactions were found for age, gender, or other comorbidities (CHF, DM).

## 4 Discussion

This large multicenter retrospective cohort study demonstrates that in-hospital BMI change is a significant and independent predictor of 28-day ICU mortality among surgical ICU patients admitted via the ED. We identified a clear non-linear, U-shaped relationship between BMI change and mortality risk, with the lowest mortality observed at a BMI reduction of approximately 1.75 kg/m^2^. Moreover, comparative analyses of body-mass metrics revealed that dynamic BMI change during hospitalization offers superior predictive performance for 28-day mortality compared with static weight or BMI measurements at either admission or discharge, underscoring its potential utility in clinical risk assessment for this vulnerable population.

In the present study, the observed 28-day mortality was 4.7%, closely resembling the 5.42% reported by Falcão et al. (2019) in surgical ICU patients (6), thereby reinforcing the external validity of our findings. To our knowledge, mortality in surgical ICUs is generally lower than in medical or mixed ICUs, primarily because of differences in case-mix: the latter cohorts are dominated by older patients with multiple comorbidities (23, 24). When stratified by quartiles of in-hospital BMI change (Q1 to Q4), a clear gradient in mortality risk was observed, rising from 3.77% in Q1 (greatest BMI decrease) to 7.27% in Q4 (greatest BMI increase), as detailed in Table 1. This mortality rate is comparatively lower than that reported in several previous studies focusing on mixed or medical ICU populations. The relatively lower mortality in our study may be attributable to the specific inclusion of surgical patients admitted through the ED, who might benefit from timely surgical interventions and targeted perioperative management. Furthermore, the exclusion of patients with extreme BMI changes (exceeding mean ± 3 SD) or short ICU stays (<24 h) likely contributed to a more stable cohort with a different risk profile compared to general ICU populations.

The relationship between baseline body composition, as crudely estimated by admission BMI, and mortality in critically ill patients remains complex and at times paradoxical. While some studies, particularly in medical ICU populations, have reported the existence of an “obesity paradox” where overweight or obese patients exhibit lower mortality rates compared to underweight or normal-weight individuals (25, 26), our findings in a surgical ICU cohort admitted via the ED did not identify admission BMI or discharge BMI as significant predictors of 28-day mortality in univariate analysis. Moreover, we compared the prognostic value of various BMI-related indices for 28-day mortality in surgical ICU patients and found that in-hospital BMI change exhibited the highest discriminatory performance, as detailed in Supplementary Table S4. This suggests that a single, static measure of body composition at a single time point may be insufficient to capture the dynamic metabolic and fluid shifts that truly impact prognosis in acute surgical settings (27). In contrast, our study establishes that the change in BMI during hospitalization is a robust and independent predictor of mortality. Even after comprehensive adjustment, the positive association persisted. Patients in the highest quartile of BMI increase (Q4) faced a 52% greater risk of mortality compared to those in the lowest quartile (Q1). This result is consistent with a growing body of literature linking positive fluid balance and weight gain during ICU stay to impaired organ function and increased mortality (28, 29), likely reflecting pathological fluid overload rather than anabolic gain. The discrepancy between our findings and those reporting the obesity paradox may be attributed to key differences in study population—specifically, the inclusion of emergency surgical patients experiencing acute physiological stress—and, most importantly, the focus on dynamic change rather than a static baseline measurement, which more accurately captures the deleterious impact of fluid accumulation and metabolic dysregulation on survival (30).

To further elucidate the true nature of the association between in-hospital BMI change and 28-day mortality, we employed restricted cubic spline (RCS) analysis. The results revealed a significant non-linear U-shaped relationship (P for non-linearity < 0.001). This finding contrasts sharply with the linear association reported by Zhang et al. in their study using the same database, which suggested that any increase in BMI was associated with increased mortality risk (31). Threshold analysis precisely identified an inflection point at −1.75 kg/m^2^, indicating the lowest mortality risk around this moderate degree of weight reduction. Mortality risk increased sharply when BMI rose above this threshold, while showing a gradual increase when BMI decrease exceeded −1.75 kg/m^2^ (Table 3). This U-shaped pattern aligns well with findings from critical illness fluid balance studies. Shen Y et al. (32) demonstrated that achieving negative fluid balance (or mild weight reduction) through deresuscitation strategies in the later phases of critical illness was associated with improved survival, potentially through mitigating organ dysfunction related to fluid overload. Concurrently, our observation that significant weight gain (right arm of the U-curve) correlates with higher mortality is supported by extensive literature linking positive fluid balance with adverse outcomes. The optimal BMI reduction identified in our study (approximately 1.75 kg/m^2^) is more pronounced than values reported in some medical ICU studies. This discrepancy primarily stems from two factors. First, the study populations differ: our cohort specifically comprised surgical patients admitted via the ED, who experience more pronounced third-space fluid shifts and greater inflammatory insults, making them particularly susceptible to fluid overload. Second, the methodological approaches differ: unlike the linear model used by Zhang et al. (31), our flexible modeling strategy (restricted cubic splines) more accurately captured the underlying non-linear relationship. Therefore, for this specific surgical population, a moderate reduction in BMI likely reflects successful fluid mobilization and is associated with optimal clinical outcomes.

The most plausible explanation for the strong association between BMI increase and mortality in our cohort is fluid overload. In critically ill surgical patients, significant weight gain over a short period is far more likely to represent pathological fluid accumulation rather than anabolic growth. This interpretation is corroborated by consistent laboratory patterns across BMI-gain quartiles: the stepwise decrease in RBC and Hb, accompanied by a concurrent rise in WBC (Table 1), aligns with haemodilution and inflammatory capillary leak—rather than blood loss or malnutrition. Patients with the greatest BMI gain (Q4) present higher illness-severity scores (APACHE IV, SOFA) and greater requirement for organ-support therapies (mechanical ventilation, dialysis), mirroring exactly the subgroup in which positive fluid balance is both most frequent and most harmful. Although the eICU-CRD contains no intake/output records, the alignment of physiologic rationale with consistent clinical and laboratory findings reinforces the inference that BMI change captures clinically meaningful fluid accumulation.

Furthermore, clinical correlates strongly support this mechanism. Patients in the highest BMI-change quartile (Q4) exhibited higher illness severity scores (APACHE IV, SOFA) and increased need for organ support (ventilation, dialysis)—clinical contexts in which fluid overload is frequently observed and deleteriously influences outcomes. Thus, our results align with a substantial body of literature linking positive fluid balance to organ dysfunction and increased mortality. Conversely, the elevated mortality observed at the left end of the U-shaped curve, where extreme BMI reduction occurs, may reflect severe catabolism and loss of lean body mass. This pattern underscores the dual interpretation of in-hospital BMI change: it serves as a marker of fluid status in the positive range and potentially of nutritional depletion in the negative range. COPD was the only subgroup showing a significant interaction (*p* = 0.037). Its systemic inflammatory state and increased capillary leak accelerate fluid accumulation, while pulmonary hyper-inflation and reduced right-ventricular preload render these patients prone to right-heart failure and increased work of breathing even after mild fluid loading (33, 34). Consequently, for any given rise in BMI, mortality climbs more steeply in COPD patients.

This study possesses several notable methodological strengths and offers significant clinical implications. First, it is a large multinational cohort study utilizing data from multiple centers, which enhances the generalizability and statistical power of our findings. Second, we employed multiple imputation to handle missing data, reducing potential bias and preserving the integrity of the dataset. Third, the association remained robust after extensive adjustment for a comprehensive set of clinically relevant covariates, including demographics, and severity scores, strengthening the validity of our conclusions. Fourth, we conducted detailed subgroup analyses and tested for interactions, which demonstrated the consistency of the association across various patient strata and increased the granularity of our results. Fifth, the identification of a specific, clinically actionable threshold for BMI change provides a potential target for goal-directed fluid and nutritional management in this population. These findings suggest that monitoring in-hospital BMI dynamics could serve as a simple, practical tool for improving risk stratification and guiding personalized care strategies for emergently admitted surgical ICU patients.

This study has several limitations. First, the primary exposure, in-hospital BMI change, is interpreted as a surrogate for fluid balance, a limitation inherent to databases lacking direct fluid intake/output documentation. Although the clinical and laboratory correlates strongly support this interpretation, we cannot definitively partition the observed weight change into its fluid, catabolic, and anabolic components. The database does not capture nutrition route, surgical complexity, or specific fluid protocols. We partially adjusted for these factors by including glucocorticoid use, dialysis, ventilator days, and APACHE-IV (which incorporates major surgery), yet unrecorded peri-operative confounders may persist. Relatedly, the timing of the “discharge weight” measurement was not standardized, representing the last available value prior to an endpoint. Second, BMI change serves only as a surrogate for fluid balance and nutritional status and cannot distinguish whether weight variations stem from fluid retention, muscle catabolism, or alterations in fat mass. We lack body composition data (e.g., bioelectrical impedance analysis) to clarify the underlying mechanisms. Furthermore, as U. S. ICUs do not routinely perform weighing at the time of death and the eICU-CRD lacks standardized pre-discharge weighing timepoints, missing weight records may introduce survivor bias. Third, the data originate from the U. S.-based eICU database with a predominantly Caucasian population, which may limit the generalizability of our findings to other ethnic groups or healthcare systems (e.g., Asian populations). Fourth, the exclusion of patients with BMI changes exceeding 15% or ICU stays shorter than 24 h may introduce selection bias and restrict the applicability of our results to extremely critical or short-stay populations. Fifth, although ROC analysis indicated that dynamic BMI change outperformed static measures, its AUC remained modest (57.92%), suggesting that integrating additional clinical variables is necessary to enhance prognostic accuracy. It is important to emphasize that BMI does not capture acute changes in lean body mass or fluid balance; therefore, any inference regarding “nutritional status” based solely on BMI should be considered approximate and requires validation with clinical and biochemical indicators. Future studies should integrate hourly ICU fluid balance records with validated malnutrition screening tools (e.g., NRS-2002) and bedside body composition markers (e.g., mid-upper arm circumference, handgrip strength, bioelectrical impedance) to differentiate fluid-induced weight gain from true muscle loss. As this study utilized discharge BMI to calculate in-hospital change, this metric can only be generated retrospectively and cannot serve as an early predictor at ICU admission. Whether serial bedside weight measurements converted into dynamic BMI trajectories can provide real-time risk alerts warrants evaluation in prospective studies equipped with automated weighing systems and electronic alert mechanisms. Importantly, the spline-determined nadir (≈ − 1.75 kg m^−2^) is a statistical optimum, not a biologically immutable threshold. Consequently, the figure should be viewed as a risk-range indicator rather than a precise therapeutic goal. Randomized trials are required to verify whether deliberate modulation of fluid balance to this zone translates into mortality benefit.

## 5 Conclusion

This multicenter retrospective cohort study demonstrates a significant non-linear U-shaped relationship between in-hospital BMI change and 28-day mortality, with the lowest risk observed at a BMI reduction of approximately 1.75 kg/m^2^. Dynamic BMI change during hospitalization offers superior prognostic value compared with static BMI or weight measured at admission or discharge. Serial BMI monitoring can refine risk stratification and guide individualized fluid and nutritional management, thereby holding important clinical value.

## Data Availability

Publicly available datasets were analyzed in this study. This data can be found at: the datasets presented in this study can be accessed online at: https://physionet.org/content/eicu-crd/2.0/.

## Footnotes

1 www.empowerstats.com

2 http://www.r-project.org

## References

[1] Herring AA Ginde AA Fahimi J Alter HJ Maselli JH Espinola JA . Increasing critical care admissions from U.S. emergency departments, 2001-2009. Crit Care Med. (2013) 41:1197–204. doi: 10.1097/ccm.0b013e31827c086f, 23591207 PMC3756824

[2] Kim YC Kim JH Ahn JY Jeong SJ Ku NS Choi JY . Discontinuation of glycopeptides in patients with culture negative severe sepsis or septic shock: a propensity-matched retrospective cohort study. Antibiotics (Basel). (2020) 9:250. doi: 10.3390/antibiotics9050250, 32414054 PMC7277931

[3] Zhang Y Zhang J Du Z Ren Y Nie J Wu Z . Risk factors for 28-day mortality in a surgical ICU: a retrospective analysis of 347 cases. Risk Manag Healthc Policy. (2021) 14:1555–62. doi: 10.2147/RMHP.S303514, 33889038 PMC8054819

[4] Oh TK Song IA Jeon YT. Peri-operative serum lactate level and postoperative 90-day mortality in a surgical ICU: a retrospective association study. Eur J Anaesthesiol. (2020) 37:31–7. doi: 10.1097/EJA.0000000000001117, 31724965

[5] Schoe A Bakhshi-Raiez F de Keizer N van Dissel JT de Jonge E. Mortality prediction by SOFA score in ICU-patients after cardiac surgery; comparison with traditional prognostic-models. BMC Anesthesiol. (2020) 20:65. doi: 10.1186/s12871-020-00975-2, 32169047 PMC7068937

[6] Falcão ALE Barros AGA Bezerra AAM Ferreira NL Logato CM Silva FP . The prognostic accuracy evaluation of SAPS 3, SOFA and APACHE II scores for mortality prediction in the surgical ICU: an external validation study and decision-making analysis. Ann Intensive Care. (2019) 9:18. doi: 10.1186/s13613-019-0488-9 30701392 PMC6353976

[7] Ge Y Wang G Huang Y Zhang Y. Association between hemoglobin glycation index and mortality in surgical ICU patients. Sci Rep. (2025) 15:37668. doi: 10.1038/s41598-025-21524-2, 41152344 PMC12569067

[8] Kądziołka I Świstek R Borowska K Tyszecki P Serednicki W. Validation of APACHE II and SAPS II scales at the intensive care unit along with assessment of SOFA scale at the admission as an isolated risk of death predictor. Anaesthesiol Intensive Ther. (2019) 51:107–11. doi: 10.5114/ait.2019.86275, 31268271

[9] Zhang XM Zhang WW Yu XZ Dou Q-L Cheng ASK. Comparing the performance of SOFA, TPA combined with SOFA and APACHE-II for predicting ICU mortality in critically ill surgical patients: a secondary analysis. Clin Nutr. (2020) 39:2902–9. doi: 10.1016/j.clnu.2019.12.026, 32008873

[10] Prentice AM Jebb SA. Beyond body mass index. Obes Rev. (2001) 2:141–7. doi: 10.1046/j.1467-789x.2001.00031.x, 12120099

[11] Xu D Lu Y Wang Y Li F. The obesity paradox and 90 day mortality in chronic critically ill patients: a cohort study using a large clinical database. Eur J Med Res. (2024) 29:329. doi: 10.1186/s40001-024-01962-w 39075583 PMC11285416

[12] Seko Y Kato T Morimoto T Yaku H Inuzuka Y Tamaki Y . Association between body mass index and prognosis of patients hospitalized with heart failure. Sci Rep. (2020) 10:16663. doi: 10.1038/s41598-020-73640-w 33028856 PMC7542148

[13] Kaplan JM Nowell M Lahni P Shen H Shanmukhappa SK Zingarelli B. Obesity enhances sepsis-induced liver inflammation and injury in mice. Obesity (Silver Spring). (2016) 24:1480–8. doi: 10.1002/oby.21504, 27172993 PMC4925204

[14] Ha L Hauge T Iversen PO. Body composition in older acute stroke patients after treatment with individualized, nutritional supplementation while in hospital. BMC Geriatr. (2010) 10:75. doi: 10.1186/1471-2318-10-75, 20955603 PMC2965141

[15] Shiao CC Huang YT Lai TS Huang T-M Wang J-J Huang C-T . Perioperative body weight change is associated with in-hospital mortality in cardiac surgical patients with postoperative acute kidney injury. PLoS One. (2017) 12:e0187280. doi: 10.1371/journal.pone.0187280, 29149189 PMC5693407

[16] Hall-Angerås M Angerås U Zamir O Hasselgren PO Fischer JE. Interaction between corticosterone and tumor necrosis factor stimulated protein breakdown in rat skeletal muscle, similar to sepsis. Surgery. (1990) 108:460–6. 2382237

[17] Hall A Dixon A Ostermann M Crichton S Skorniakov I Kellum JA. Fluid removal associates with better outcomes in critically ill patients receiving continuous renal replacement therapy: a cohort study. Crit Care. (2020) 24:279. doi: 10.1186/s13054-020-02986-4, 32487189 PMC7268712

[18] Fan H Yuan Z Zhou J Huang Y Zhang H Feng X. Association of four nutritional scores with all-cause and cardiovascular mortality in the general population. Front Nutr. (2022) 9:846659. doi: 10.3389/fnut.2022.846659, 35433793 PMC9006821

[19] Pollard TJ Johnson AEW Raffa JD Celi LA Mark RG Badawi O. The eICU collaborative research database, a freely available multi-center database for critical care research. Sci Data. (2018) 5:180178. doi: 10.1038/sdata.2018.178, 30204154 PMC6132188

[20] Theander L Willim M Nilsson JÅ Karlsson M Åkesson KE Jacobsson LTH . Changes in bone mineral density over 10 years in patients with early rheumatoid arthritis. RMD Open. (2020) 6:e001142. doi: 10.1136/rmdopen-2019-001142, 32519976 PMC7046965

[21] Zhang L Liu H Zhang D Deng Y Chen X Zhang L. Non-linear association between lactate and 28-day mortality in elderly patients with sepsis across different SOFA score groups: results from the eICU collaborative research database. Front Med. (2025) 12:1605319. doi: 10.3389/fmed.2025.1605319 PMC12267231 40678131

[22] Groenwold RH White IR Donders AR Carpenter JR Altman DG Moons KG. Missing covariate data in clinical research: when and when not to use the missing-indicator method for analysis. CMAJ. (2012) 184:1265–9. doi: 10.1503/cmaj.110977, 22371511 PMC3414599

[23] Wang G Liu P Xie H Niu C Lyu J An Y . Impact of glucocorticoid therapy on 28-day mortality in patients having severe fever with thrombocytopenia syndrome in an intensive care unit: a retrospective analysis. J Inflamm Res. (2024) 17:7627–37. doi: 10.2147/JIR.S478520, 39479263 PMC11521778

[24] Thibault R Makhlouf AM Mulliez A Cristina Gonzalez M Kekstas G Kozjek NR . Fat-free mass at admission predicts 28-day mortality in intensive care unit patients: the international prospective observational study phase angle project. Intensive Care Med. (2016) 42:1445–53. doi: 10.1007/s00134-016-4468-3, 27515162

[25] Polemiti E Baudry J Kuxhaus O Jäger S Bergmann MM Weikert C. BMI and BMI change following incident type 2 diabetes and risk of microvascular and macrovascular complications: the EPIC-Potsdam study. Diabetologia. (2021) 64:814–825. doi: 10.1007/s00125-020-05362-7, 33452586 PMC7940263

[26] Pepper DJ Sun J Welsh J Cui X Suffredini AF Eichacker PQ. Increased body mass index and adjusted mortality in ICU patients with sepsis or septic shock: a systematic review and meta-analysis. Crit Care. (2016) 20:181. doi: 10.1186/s13054-016-1360-z, 27306751 PMC4908772

[27] Liu W Zeng W Huang Z Yuan Q. Association of body mass index changes with short-term mortality risks in ICU patients with sepsis across different admission BMI states: analysis of the MIMIC-IV database. Front Immunol. (2025) 16:1698405. doi: 10.3389/fimmu.2025.1698405, 41209005 PMC12591966

[28] Messmer AS Zingg C Müller M Gerber JL Schefold JC Pfortmueller CA. Fluid overload and mortality in adult critical care patients—a systematic review and meta-analysis of observational studies. Crit Care Med. (2020) 48:1862–70. doi: 10.1097/CCM.0000000000004617, 33009098

[29] Chandra J Armengol de la Hoz MA Lee G Lee A Thoral P Elbers P . A novel vascular leak index identifies sepsis patients with a higher risk for in-hospital death and fluid accumulation. Crit Care. (2022) 26:103. doi: 10.1186/s13054-022-03968-4, 35410278 PMC9003991

[30] Boyd JH Forbes J Nakada TA Walley KR Russell JA. Fluid resuscitation in septic shock: a positive fluid balance and elevated central venous pressure are associated with increased mortality. Crit Care Med. (2011) 39:259–65. doi: 10.1097/CCM.0b013e3181feeb15, 20975548

[31] Zhang J Du L Jin X Ren J Li R Liu J . Association between body mass index change and mortality in critically ill patients: a retrospective observational study. Nutrition. (2023) 105:111879. doi: 10.1016/j.nut.2022.111879, 36413821

[32] Shen Y Zhang W Shen Y. Early diuretic use and mortality in critically ill patients with vasopressor support: a propensity score-matching analysis. Crit Care. (2019) 23:9. doi: 10.1186/s13054-019-2309-9, 30630521 PMC6329160

[33] Barnes PJ Celli BR. Systemic manifestations and comorbidities of COPD. Eur Respir J. (2009) 33:1165–85. doi: 10.1183/09031936.00128008, 19407051

[34] Laghi F Tobin MJ. Disorders of the respiratory muscles. Am J Respir Crit Care Med. (2003) 168:10–48. doi: 10.1164/rccm.2206020, 12826594

