# Supplementary material for: Impact of in-hospital body mass index variation on 28-day mortality in critically ill surgical patients: a multi-center retrospective analysis
Source: Front Med (Lausanne). 2026 Jan 27;12:1738654. doi: 10.3389/fmed.2025.1738654 (PMC12886406; doi:10.3389/fmed.2025.1738654)
Supplement: Supplementary file 1 [file Table_1.DOCX]

**Impact of in-hospital body mass index variation on 28-day mortality in critically ill surgical patients: a multi-center retrospective analysis**

**Bocheng Yang^1†^, Gang Xu^2†^, Jiagui Zhao ^3^**^†^, **Xiaoan Yang^4*^, Qinghe Huang^5*^**

1. Division of Plastic Surgery, Zhongshan Hospital Xiamen University, School of Medicine, Xiamen University, Xiamen 361004, China;

2. Medical Emergency Center of Xiamen,Xiamen,Chian

3. Department of Critical Care Medicine, Zhongda Hospital, School of Medicine, Southeast University, Jiangsu, 210009, China

4. Department of Infectious Diseases, the 3rd Affiliated Hospital, Sun Yat‑Sen University, No 600, Tianhe road, Tianhe district, Guangzhou, 510630, China.

5. Department of ICU, Zhongshan Hospital of Xiamen University, School of Medicine, Xiamen University, Xiamen 361004, China;

†Bocheng Yang, Jiagui Zhao and Gang Xu contributed equally to this work.

*Corresponding authors:

**Xiaoan Yang**

Department of Infectious Diseases, the 3rd Affiliated Hospital, Sun Yat‑Sen University, No 600, Tianhe road, Tianhe district, Guangzhou, 510630, China. Email: yxan@mail2.sysu.edu.cn

**Qinghe Huang**

Department of ICU, Zhongshan Hospital of Xiamen University, School of Medicine, Xiamen University, Xiamen 361004, China. Email: huang_qinghe@126.com

**Fund:**

This study was supported by 2024 Provincial Natural Science Foundation of Joint Funding Project (No.2024J0113342) and Xiamen Municipal Natural Science Foundation Project (No.3502Z202374016).

**Running title:** **BMI Change on 28-Day Mortality**

**Key words:** BMI change, 28-day mortality, Surgical ICU, Emergency department admission, U-shaped relationship

**TABLE S1 Baseline Characteristics Before and After Multiple Imputation.**

| Characteristics | Before MI (N=20543) | After MI (N=20543) | P-value |  |
| --- | --- | --- | --- | --- |
| Age（years） | 62.50 ± 17.45 | 62.50 ± 17.45 | 0.998 |  |
| Admission BMI (kg/m2) | 28.81 ± 8.34 | 28.81 ± 8.34 | 1.000 |  |
| Discharge BMI (kg/m2) | 29.03 ± 8.35 | 29.03 ± 8.35 | 0.999 |  |
| BMI change (kg/m2) | 0.22 ± 1.99 | 0.22 ± 1.99 | 0.998 |  |
| Gender |  |  | 1.000 |  |
| Female, n (%) | 9674 (47.09) | 9674 (47.09) |  |  |
| Male, n (%) | 10869 (52.91) | 10869 (52.91) |  |  |
| GCS score | 12.57 ± 3.65 | 12.56 ± 3.66 | 0.773 |  |
| APACHE IV score | 57.55 ± 25.57 | 57.55 ± 25.57 | 1.000 |  |
| SOFA score | 3.12 ± 2.72 | 3.12 ± 2.72 | 0.999 |  |
| Laboratory tests |  |  |  |  |
| BUN, (mmol/L) | 27.07 ± 23.05 | 26.41 ± 22.58 | 0.404 |  |
| Scr （mg/dL） | 1.55 ± 1.79 | 1.52 ± 1.76 | 0.080 |  |
| Serum calcium, (mmol/L） | 8.33 ± 0.80 | 8.34 ± 0.79 | 0.38 |  |
| Serum K, (mmol/L） | 4.08 ± 0.72 | 4.08 ± 0.71 | 0.518 |  |
| GLU, (mg/dl) | 152.80 ± 96.27 | 151.37 ± 94.49 | 0.138 |  |
| AST, (U/L) | 134.10 ± 654.79 | 119.11 ± 604.92 | 0.051 |  |
| ALT, (U/L) | 87.03 ± 351.23 | 79.48 ± 334.58 | 0.074 |  |
| PLT, (×10^9^/L) | 206.90 ± 94.16 | 208.17 ± 94.75 | 0.191 |  |
| RBC, (×10^12^/L) | 3.79 ± 0.79 | 3.79 ± 0.79 | 0.570 |  |
| HBG (g/L) | 11.27 ± 2.41 | 11.30 ± 2.40 | 0.289 |  |
| RDW, (%) | 15.22 ± 2.47 | 15.19 ± 2.45 | 0.316 |  |
| WBC, (×10^9^/L) | 11.95 ± 10.87 | 11.81 ± 10.35 | 0.207 |  |
| Comorbidities |  |  |  |  |
| COPD, n (%) | 1933 (9.41) | 1933 (9.41) | 1.000 |  |
| CHF, n (%) | 1821 (8.86) | 1821 (8.86) | 1.000 |  |
| AMI, n (%) | 1136 (5.53) | 1136 (5.53) | 1.000 |  |
| DM, n (%) | 5608 (27.30) | 5607 (27.29) | 0.736 |  |
| Vent, n (%) | 5300 (25.80) | 5300 (25.80) | 1.000 |  |
| Intubated, n (%) | 3058 (14.89) | 3058 (14.89) | 1.000 |  |
| Dialysis, n (%) | 670 (3.26) | 670 (3.26) | 1.000 |  |
| 28-day ICU mortality |  |  | 1.000 |  |
| No, n (%) | 19577 (95.30) | 19577 (95.30) |  |  |
| Yes, n (%) | 966 (4.70) | 966 (4.70) |  |  |

Continuous variables are summarized as mean (SD) or median (trisection interval); categorical variables are presented as percentages (%). BMI, body mass index; COPD, chronic obstructive pulmonary disease; AMI, acute myocardial infarction; DM, diabetes mellitus; CHF, Congestive Heart Failure; ICU, intensive care unit; LOS, length of stay; BUN, blood urea nitrogen; RBC, red blood cell; HGB, hemoglobin; PLT, platelets; GLU, glucose; AST, aspartate aminotransferase; ALT, alanine aminotransferase; RDW, red cell distribution width; WBC, white blood cells; GCS score, glasgow coma scale; APACHE IV, acute physiology and chronic health evaluation IV; SOFA score: sequential organ failure assessment.

**TABLE S2 Factors influencing risk of 28-day ICU mortality analyzed by univariate Cox proportional hazards regression analysis**

| Variable | Statistics | HR (95 CI) P-value |
| --- | --- | --- |
| Age（years） | 62.50 ± 17.45 | 1.02 (1.01, 1.02) <0.0001 |
| Admission BMI (kg/m2) | 28.81 ± 8.34 | 0.99 (0.99, 1.00) 0.1152 |
| Discharge BMI (kg/m2) | 29.03 ± 8.35 | 1.00 (1.00, 1.01) 0.5321 |
| BMI change (kg/m2) | 0.22 ± 1.99 | 1.10 (1.08, 1.13) <0.0001 |
| **Gender** |  |  |
| Female, n (%) | 9674 (47.09%) | 1.0 |
| Male, n (%) | 10869 (52.91%) | 0.96 (0.85, 1.09) 0.5277 |
| **Ethnicity** |  |  |
| Caucasian, n (%) | 16236 (79.03%) | 1.0 |
| African-American, n (%) | 2325 (11.32%) | 0.87 (0.71, 1.07) 0.1995 |
| Hispanic, n (%) | 789 (3.84%) | 0.77 (0.55, 1.08) 0.1247 |
| Asian, n (%) | 390 (1.90%) | 0.81 (0.51, 1.28) 0.3667 |
| Native American, n (%) | 220 (1.07%) | 1.47 (0.87, 2.50) 0.1506 |
| Unknown, n (%) | 583 (2.84%) | 0.81 (0.54, 1.23) 0.3200 |
| GCS score | 12.56 ± 3.66 | 0.87 (0.85, 0.88) <0.0001 |
| APACHE IV score | 57.55 ± 25.57 | 1.03 (1.03, 1.03) <0.0001 |
| SOFA score | 3.12 ± 2.72 | 1.25 (1.23, 1.27) <0.0001 |
| Vent, n (%) | 5300 (25.80%) | 2.48 (2.17, 2.83) <0.0001 |
| Intubated, n (%) | 3058 (14.89%) | 2.32 (2.03, 2.64) <0.0001 |
| Dialysis, n (%) | 670 (3.26%) | 1.64 (1.23, 2.19) 0.0007 |
| Glucocorticoid use | 82 (0.40%) | 1.35 (0.50, 3.59) 0.5539 |
| **Laboratory tests** | 4.10 ± 0.75 | 1.21 (1.11, 1.33) <0.0001 |
| BUN, median (IQR), (mmol/L) | 19.00 (12.00-32.00) | 1.01 (1.01, 1.01) <0.0001 |
| Scr (mg/dL) | 1.00 (0.73-1.55) | 1.07 (1.04, 1.09) <0.0001 |
| Serum calcium， （mmol/L） | 8.34 ± 0.79 | 0.75 (0.70, 0.81) <0.0001 |
| Serum K， （mmol/L） | 4.08 ± 0.71 | 1.28 (1.19, 1.38) <0.0001 |
| GLU, median (IQR), (mg/dl) | 151.37 ± 94.49 | 1.00 (1.00, 1.00) <0.0001 |
| AST, (U/L) | 28.00 (17.00-58.00) | 1.00 (1.00, 1.00) <0.0001 |
| ALT, (U/L) | 26.00 (16.00-45.00) | 1.00 (1.00, 1.00) <0.0001 |
| PLT, (×10^9^/L) | 208.17 ± 94.75 | 1.00 (1.00, 1.00) <0.0001 |
| RBC, (×10^12^/L) | 3.79 ± 0.79 | 0.91 (0.84, 0.98) 0.0134 |
| HBG (g/L) | 11.30 ± 2.40 | 0.97 (0.94, 0.99) 0.0106 |
| RDW, (%) | 15.19 ± 2.45 | 1.10 (1.08, 1.12) <0.0001 |
| WBC, (×10^9^/L) | 11.81 ± 10.35 | 1.01 (1.01, 1.01) <0.0001 |
| **Comorbidities** | 2.14 (1.54-3.61) | 0.35 (0.33, 0.37) <0.0001 |
| COPD, n (%) | 1933 (9.41%) | 0.94 (0.76, 1.16) 0.5364 |
| CHF, n (%) | 1821 (8.86%) | 1.18 (0.97, 1.43) 0.0939 |
| AMI, n (%) | 1136 (5.53%) | 1.17 (0.90, 1.53) 0.2270 |
| DM, n (%) | 5607 (27.29%) | 1.01 (0.88, 1.17) 0.8418 |
| ICU LOS, median (IQR), d | 2.24 (1.58-3.79) | 0.00 (0.00, 0.00) <0.0001 |
| Hospital LOS, median (IQR), d | 4.89 (2.96-8.26) | 0.00 (0.00, 0.00) <0.0001 |

**TABLE S3.** **Association between BMI change and 28-day ICU mortality based on raw data.**

| Model | Exposure | HR (95 CI) P-value |
| --- | --- | --- |
| **Model I** | **BMI change** **as continuous** | 1.10 (1.08, 1.13) <0.0001 |
|  | Q1 | Ref |
|  | Q2 | 1.38 (1.06, 1.78) 0.0148 |
|  | Q3 | 1.87 (1.56, 2.24) <0.0001 |
|  | Q4 | 2.07 (1.74, 2.46) <0.0001 |
|  | P for trend | <0.0001 |
| **Model II** | **BMI change** **as continuous** | 1.10 (1.07, 1.13) <0.0001 |
|  | Q1 | Ref |
|  | Q2 | 1.35 (1.05, 1.75) 0.0214 |
|  | Q3 | 1.84 (1.53, 2.20) <0.0001 |
|  | Q4 | 2.04 (1.71, 2.43) <0.0001 |
|  | P for trend | <0.0001 |
| **Model III** | **BMI change** **as continuous** | 1.04 (1.00, 1.08) 0.0290 |
|  | Q1 | Ref |
|  | Q2 | 1.09 (0.75, 1.60) 0.6402 |
|  | Q3 | 1.29 (0.99, 1.68) 0.0616 |
|  | Q4 | 1.33 (1.03, 1.71) 0.0290 |
|  | P for trend | 0.0205 |

Model I: we did not account for additional variables.

Model II: we adjusted gender, age, ethnicity.

Model III: we adjusted gender, age, ethnicity, SOFA score, APACHE IV score, LOS-ICU, vent. HR, Hazard Ratios; CI, confidence; Ref, reference.

**TABLE S****4 Discriminative Ability of** **BMI change for 28-Day Mortality in ICU**

| Variable | AUC | (95CI) |
| --- | --- | --- |
| Admission weight | 49.5774% | (47.6715% ~ 51.4832%) |
| Discharge weight | 51.8196% | (49.9223% ~ 53.7169%) |
| Admission BMI | 50.6441% | (48.7317% ~ 52.5564%) |
| Discharge BMI | 52.0062% | (50.1129% ~ 53.8995%) |
| BMI change | 57.9249% | (55.9155% ~ 59.9344%) |


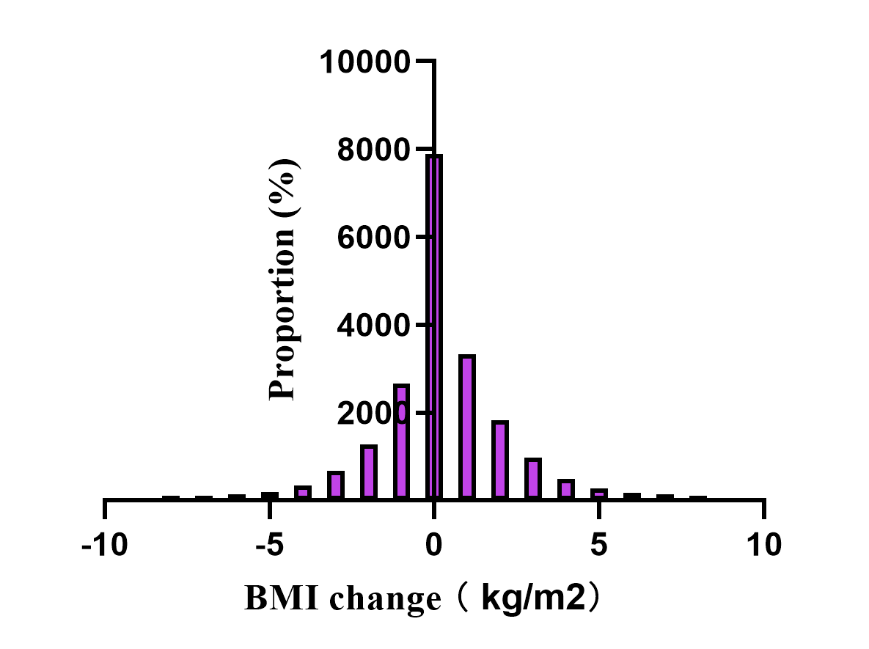


**FIGURE S1** Distribution of BMI change. It presented a normal distribution, ranging from -7.54 kg/m^2^ to 7.87 kg/m^2^, with a mean of 0.218 kg/m^2^.


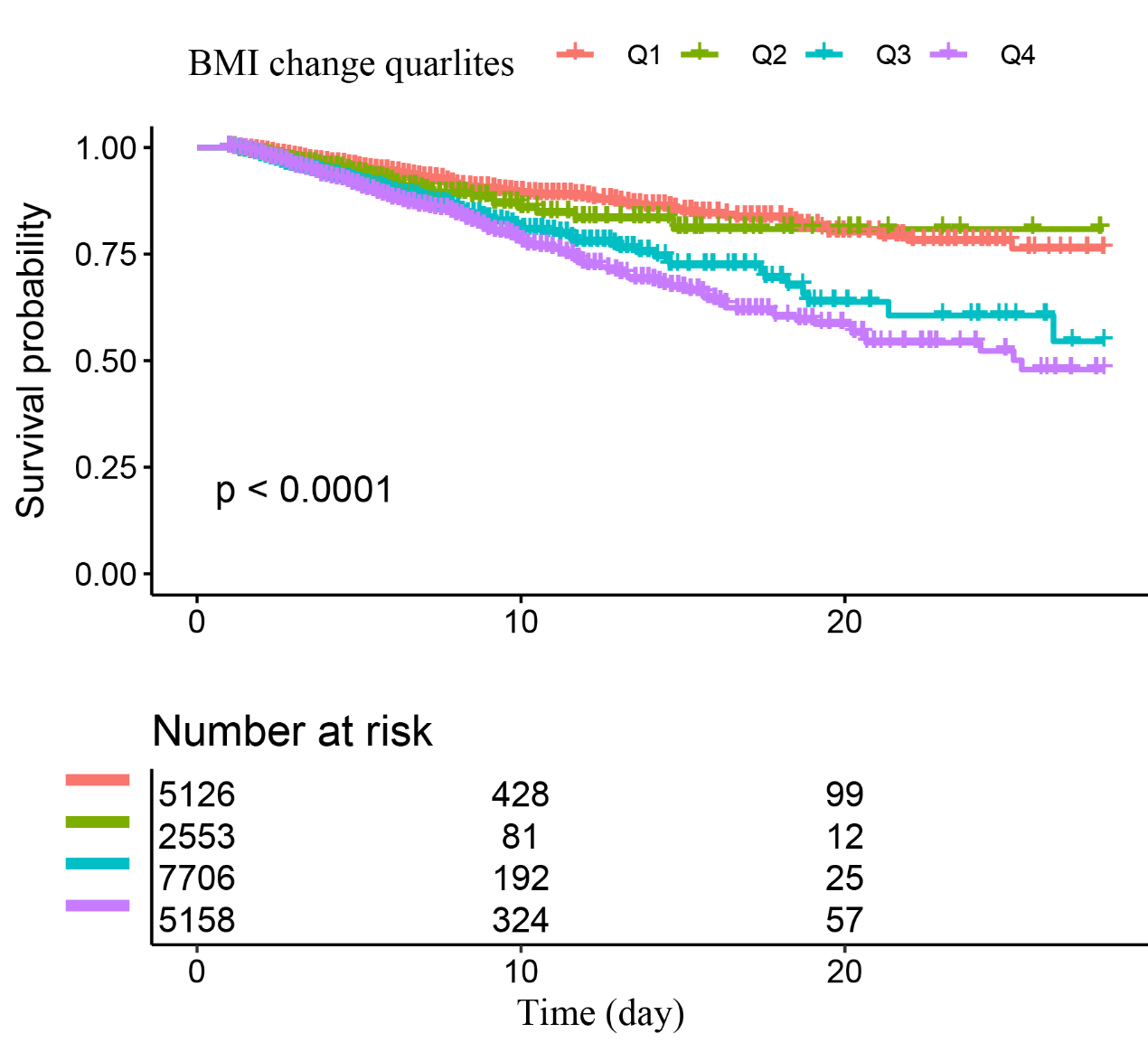


**FIGURE S2** shows the Kaplan–Meier curves for 28-day ICU mortality.


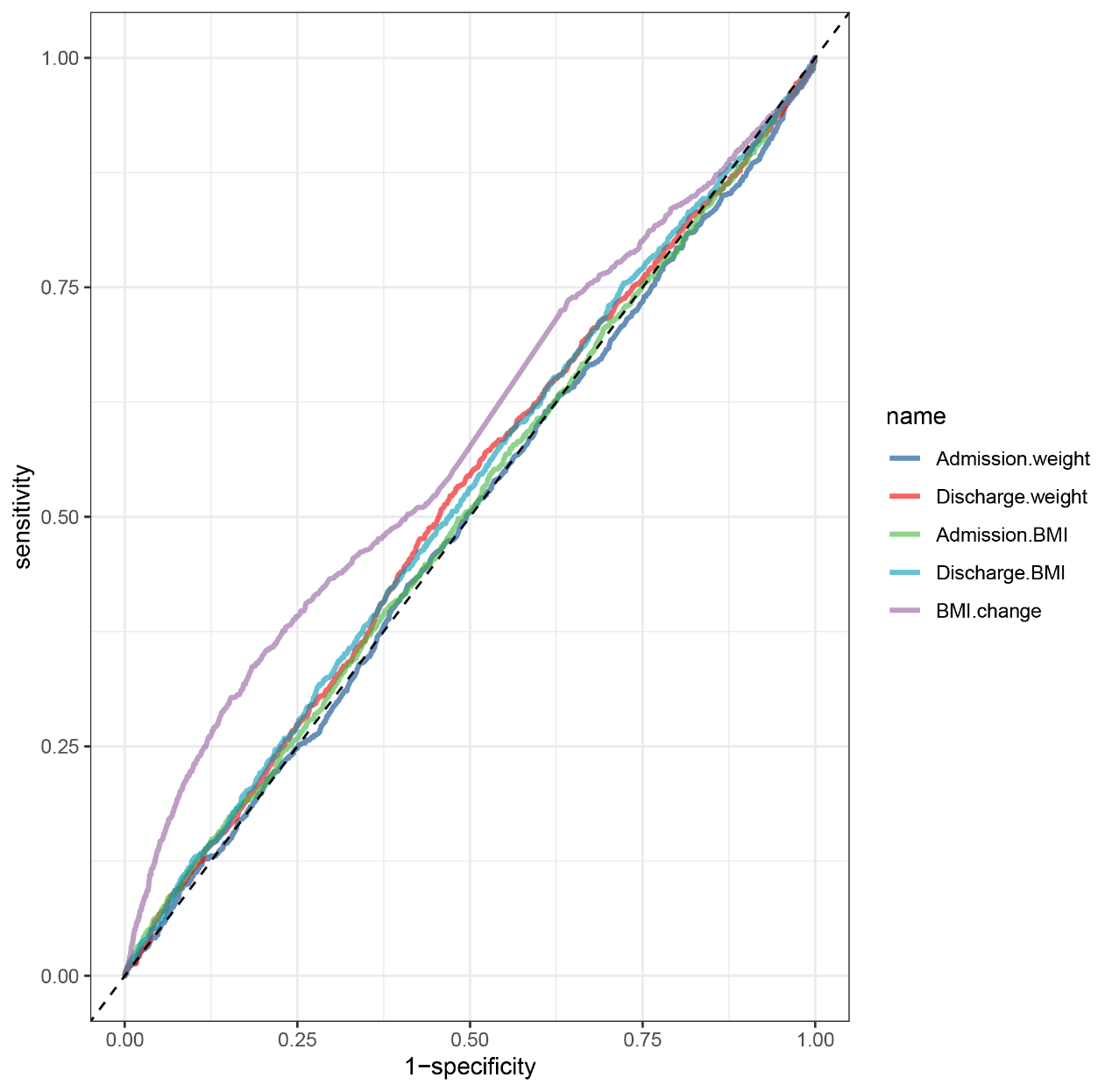


**FIGURE S3** Discriminative Performance of Body-Mass Metrics for 28-Day Mortality in ICU Patients: Receiver Operating Characteristic Curve Analysis.
